# Plantar Fascia Lateral Fascicle Rupture: How Severe Can It Be?

**DOI:** 10.7759/cureus.32987

**Published:** 2022-12-27

**Authors:** Miguel De Castro Correia, Tiago Rodrigues Lopes

**Affiliations:** 1 Physical Medicine and Rehabilitation, North Rehabilitation Center, Vila Nova de Gaia, PRT

**Keywords:** foot pain, lateral foot pain, plantar fasciitis, plantar fascia lateral fascicle, plantar fascia tear

## Abstract

The* plantar fascia* is a thick and strong group of longitudinal and transverse bands of collagen-rich tissue, consisting of central, medial, and lateral fascicles. Biomechanically, the central fascicle assumes a special role in medial longitudinal foot arch preservation. However, there is scarce data on *plantar fascia* medial and lateral fascicles' anatomy and pathology in the literature.

We report the case of a 27-year-old male professional soccer player who presented with sudden-onset, severe lateral right rearfoot pain that had started while doing linear sprinting practice. The athlete had no relevant medical history and no history of previous right foot injuries. The radiographic study of the right foot revealed no significant changes. MRI showed a high T2 signal partially interrupting the *plantar fascia* lateral fascicle low signal, consistent with a lateral fascicle rupture. The rehabilitation program was initiated and included pharmacological and non-pharmacological management. He experienced an extremely favorable evolution of his condition with the absence of pain and partial weight bearing in less than one week, with a full return to sports in approximately two weeks.

During dynamic foot movement, the lateral fascicle seems to be less biomechanically recruited compared to the central one; however, the strain is not negligible and might be the reason for the pathology. Still, this slightly decreased strain might explain this injury’s faster healing time compared to that associated with the central fascicle. Regarding the risk factors for *plantar fascia* ruptures, it should be kept in mind that a tear might occur even in their absence.

We intend to raise awareness about the existence of *plantar fascia* lateral fascicle and the possibility of associated pathology, either acute or chronic. Hopefully, in the near future, *plantar fascia* ruptures will get significantly more attention in the literature, enabling the creation of proper management guidelines.

## Introduction

The* plantar fascia* is a thick and strong group of longitudinal and transverse bands of collagen-rich tissue [1]. Its superficial fibers attach primarily to the overlying thick dermis, while most of the more extensive deep fibers originate on the medial process of the calcaneal tuberosity, course anteriorly, and insert on metatarsophalangeal joints plantar plates and flexor tendons fibrous sheaths [2], and are called *plantar fascia* central fascicles. Many authors use the term *plantar fascia *itself to refer to this central fascicle [3]. Biomechanically, this central fascicle assumes a special role in medial longitudinal foot arch preservation, especially in static situations, such as standing at ease, being this arch's primary passive support [4]. Additionally, although unknown to many, the literature has described medial and lateral *plantar fascia* fascicles [3]. The medial fascicle is the smallest in cross-section and rarely accounts for any pathology [3]. The lateral fascicle originates laterally to the *plantar fascia* center fascicle and courses laterally, inserting on the fifth metatarsal tubercle [5]. *Plantar fascia* lateral fascicle pathology is also less frequent compared to that associated with the central fascicle [3].

Even though the *plantar fascia* is frequently the reason for foot pain medical consultation, such as in *plantar fasciitis* [6], this structure tear is rarely described in the literature [6], with the first case being reported only in 1961 [7]. Moreover, even though some cases of lateral fascicle plantar fasciitis have been described [5], we did not find any report on *plantar fascia *lateral fascicle rupture. *Plantar fascia* ruptures, usually pertaining to the central fascicle, can be divided into spontaneous or acute-on-chronic, with the latter being the most frequent one [6,8]. On the one hand, spontaneous ruptures usually occur in patients without any previous symptoms and present with a popping sensation, pain on the foot plantar surface, and ecchymosis [6]. On the other hand, patients with acute-on-chronic ruptures usually have a history of *plantar fasciitis*, with many patients reporting previous corticosteroid injections, as well as milder symptoms compared to spontaneous rupture [6,8]. In both cases, patients are usually middle-aged males with a high-level, or even professional, athletic activity [6,8]. These ruptures can be partial or complete [9] and the mechanism described is an acceleration type of motion [10].

The diagnosis is usually clinical with MRI employed in differential diagnosis and to properly characterize the injury [8]. The typical MRI finding is a high T2 signal partially or completely interrupting the low signal of the *plantar fascia *[8]. As for the treatment of *plantar fascia* rupture, no formal guidelines currently exist; however, many authors have suggested that minimum possible immobilization, pain-adapted weight bearing, thrombosis prophylaxis, non-steroidal anti-inflammatory drugs (NSAIDs), ice, and physiotherapy play an important role, as stated in a systematic review by Debus et al. [6]. Most cases result in good outcomes with conservative management [8]. However, the literature has reported surgical treatment in patients who remain symptomatic after conservative management, with fibroblastic tissue excision and fascia release [8]. We discuss the case of *plantar fascia* lateral fascicle spontaneous rupture in a 27-year-old male professional soccer player that was successfully managed with conservative measures.

## Case presentation

We report the case of a 27-year-old male professional soccer player of the Portuguese ‘Primeira Liga’, a right-footer and right winger, who presented with sudden-onset, severe, lateral right rearfoot pain that had started while doing linear sprinting practice. The athlete had no relevant medical history and no history of previous right foot injuries. The athlete was promptly evaluated by the medical team, and he described a popping sensation while running, as well as a sharp pain on the lateral plantar and lateral sides of the right rear foot, with no irradiation. He scored 8 out of 10 on the Numeric Rating Scale (NRS) and had severe gait impairment, even with partial weight bearing.

On physical examination, there was a normal alignment of the ankle-foot axis, with no anterior or posterior instability and no significant edema or hematoma of the rearfoot. Passive and active ranges of motion were preserved; however, the athlete had pain associated with the overall mobility of the right foot. He reported tenderness on lateral rearfoot palpation, but there was no pain related to other tendinous, ligamentous, or bony structures. The gait cycle was severely impaired with a reduction of the right foot stance phase and a preferential midfoot and forefoot floor strike.

**Figure 1:**
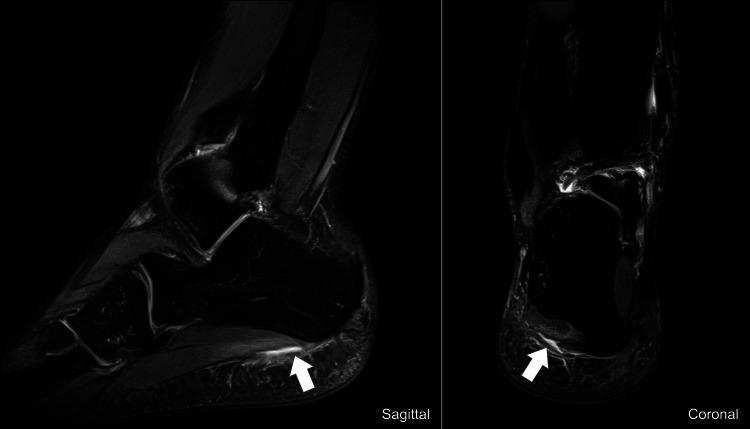
Sagittal and coronal cuts of T2-weighted MRI Note the high signal interrupting the *plantar fascia* low signal (white arrow) MRI: magnetic resonance imaging

The radiographic study of the right foot (anteroposterior and oblique view), performed on the same day of the injury, revealed no significant changes. Two days after the injury, the athlete underwent an MRI, which showed a high T2 signal partially interrupting the *plantar fascia* lateral fascicle low signal (Figure 1).

As soon as the diagnosis was established, the rehabilitation program was started, which included pharmacological and non-pharmacological management (physiotherapy and daily activities behavioral adaptations). The primary focus was pain reduction. On the first two days after injury, the athlete was prescribed 500 mg of naproxen twice daily. On the following days, 1 g of paracetamol was prescribed on demand to a maximum of 4 g daily (a minimum interval of six hours between each intake). Regarding physiotherapy, the athlete had one-hour sessions, twice daily with the same therapist. Each session started with transcutaneous electrical nerve stimulation (TENS) at 100 Hz, 200 us per pulse, and paresthesia-inducing intensity in the right rear foot for transitory analgesia, followed by manual therapy for fluid drainage and active range of motion maintenance. Pulsed therapeutic ultrasound was added with 20% of duty cycle, 3 MHz, 1 W/cm^2^ for five minutes. The athlete was allowed to perform lower limb strength exercises and aerobic training (cycling), below the pain threshold. Walking with crutches to avoid weight bearing was introduced subsequently.

The athlete experienced an extremely favorable evolution of his condition with the absence of pain and partial weight bearing in less than one week, with a full return to sports in approximately two weeks.

## Discussion

As stated above, the *plantar fascia* has a medial, central, and lateral fascicle, with most of the literature referring to the central one as the *plantar fascia* itself [3]. The mechanism of injury usually described for *plantar fascia* rupture is an acceleration type of motion [10]. As described by Neumann et al. [2], during the terminal stance phase of the gait cycle, the medial longitudinal foot arch is slightly raised for kinematic purposes, due to the “windlass effect”. As the metatarsophalangeal joints are fully extended, *plantar fascia* (central fascicle) strain increases, marginally shortening its length and elevating the medial longitudinal arch by an average of 6 mm [11]. Probably, this *plantar fascia* tension increase is responsible for the rupture's occurrence.

There are scarce data in the literature on *plantar fascia* lateral fascicle, not only pertaining to its biomechanical role but also regarding the possible mechanism behind ruptures. The lateral fascicle has a close relationship with the foot lateral column, originating in the calcaneal tuberosity, and inserting on the fifth metatarsal head tubercle, being shorter and narrower than the central fascicle [3]. Although both medial and lateral longitudinal foot arches have load-sharing capabilities, extensive biomechanical analysis of the lateral foot arch is lacking to describe any height variation during the gait cycle [12]. Thus, one could hypothesize that during the gait cycle, *plantar fascia* lateral fascicle tension is lower compared to that of the central fascicle, accounting for fewer injuries. Nonetheless, the foot lateral arch plays a role in energy conservation, stability, propulsion, and shock absorption [12], which might explain lateral fascicle fasciitis and even rupture. Therefore, although it is less biomechanically recruited compared to the central fascicle, the lateral fascicle seems to suffer significant strain during dynamic feet movement.

Based on the rationale in the previous paragraph, one could expect lateral fascicle ruptures to heal faster compared to those of the central fascicle. Although literature describes a large span for those experiencing *plantar fascia* central fascicle ruptures to return to sport (RTP), ranging from three to 64 weeks, the average RTP exceeds three months [6]. On the contrary, our athlete returned to his pre-injury sporting level in approximately two weeks (we recommend using our protocol in studies for further validation). On the one hand, this fact could favor what seems to be a less biomechanical demand for the lateral fascicle, allowing it to heal faster. On the other hand, one could question if this athlete really had a *plantar fascia* lateral fascicle rupture given his quick RTP. In fact, some authors report that ultrasound is superior to MRI in differentiating true fiber interruption and tearing from edema [8]. Therefore, in future cases, we recommend complementing MRI with ultrasound, to enable proper diagnoses and accurately define lateral fascicle healing time.

Regarding the risk factors for *plantar fascia* rupture, most authors agree that previous *plantar fasciitis* and corticosteroid injections seem to increase the risk for this pathology [6,8]. Additionally, this injury happens more frequently in middle-aged males with high-level or even professional sports activity [6,8]. However, our athlete did not report any previous injury to the injured or contralateral foot, nor any lower limb injury. Therefore, while the risk factors might be seen as possible facilitators, the condition can occur without them.

## Conclusions

The* plantar fascia* is a frequent cause of medical consultation, such as in *plantar fasciitis*. However, *plantar fascia *ruptures are drastically less common. Moreover, the literature seems to underreport medial and lateral *plantar fascia *fascicles and their inherent pathology. During dynamic foot movement, the lateral fascicle seems to be less biomechanically recruited compared to the central one; however, the strain is not negligible and might be the reason for pathology. Still, this slightly decreased strain might explain this injury’s faster healing time compared to that associated with the central fascicle. Regarding risk factors for *plantar fascia* ruptures, one should keep in mind that a tear might occur even in their absence.

To our knowledge, this is the first case that clearly identifies a *plantar fascia* lateral fascicle rupture, thereby highlighting the relevance of this structure pathology. Nonetheless, it is important to remember that we were able to reach this diagnosis because our athlete is a professional soccer player, which prompted the performance of an MRI two days after the injury. Most nonprofessional athletes or regular hospital patients are not made to undergo an MRI until a few weeks have passed after the injury. If so, and if every lateral fascicle rupture heals in two weeks - as it did in our patient - most cases will go undiagnosed. Furthermore, even after our athlete was pain-free, MRI should be repeated for comparison.

With this case report, we intend to raise awareness about the existence of *plantar fascia* lateral fascicle and the possibility of it being the cause of pathology, either acute or chronic. Hopefully, in the near future, literature will come to include a significant number of studies on *plantar fascia *ruptures, enabling the framing of proper management guidelines.
